# Relevance of Essential Trace Elements in Nutrition and Drinking Water for Human Health and Autoimmune Disease Risk

**DOI:** 10.3390/nu12072074

**Published:** 2020-07-13

**Authors:** Daniela Cannas, Eleonora Loi, Matteo Serra, Davide Firinu, Paolo Valera, Patrizia Zavattari

**Affiliations:** 1 Department of Biomedical Sciences, Unit of Biology and Genetics, University of Cagliari, 09042 Cagliari, Italy; cannasdaniela@libero.it (D.C.); eleonora.loi@unica.it (E.L.); 2 Department of Civil, Environmental Engineering and Architecture, University of Cagliari, 09123 Cagliari, Italy; srr.mtt.sa@gmail.com; 3 Department of Medical Sciences and Public Health, Monserrato Campus, University of Cagliari, 09042 Cagliari, Italy; davide.firinu@unica.it

**Keywords:** trace elements, metals, metalloids, autoimmunity, autoimmune diseases, multiple sclerosis, type 1 diabetes, elements legal regulation, elements in water and food, water policy

## Abstract

Trace elements produce double-edged effects on the lives of animals and particularly of humans. On one hand, these elements represent potentially toxic agents; on the other hand, they are essentially needed to support growth and development and confer protection against disease. Certain trace elements and metals are particularly involved in humoral and cellular immune responses, playing the roles of cofactors for essential enzymes and antioxidant molecules. The amount taken up and the accumulation in human tissues decisively control whether the exerted effects are toxic or beneficial. For these reasons, there is an urgent need to re-consider, harmonize and update current legislative regulations regarding the concentrations of trace elements in food and in drinking water. This review aims to provide information on the interrelation of certain trace elements with risk of autoimmune disease, with a particular focus on type 1 diabetes and multiple sclerosis. In addition, an overview of the current regulations and regulatory gaps is provided in order to highlight the importance of this issue for everyday nutrition and human health.

## 1. Introduction

**Figure 1:**
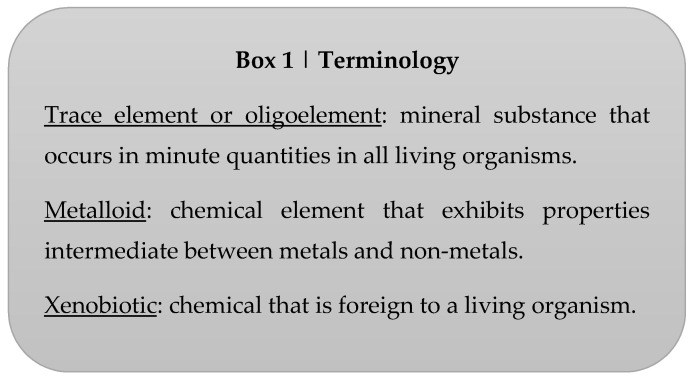
Specific terminology frequently used in the text.

The onset of different diseases, including autoimmune, metabolic, neurodegenerative diseases and cancer, has been correlated with metals, metalloids and the excess or deficiency of essential oligoelements in the body (Figure 1). These elements are ubiquitous and the body is constantly exposed to them *via* their presence in soil, water, air and food [1,2,3].

Many metals are essential to numerous biological, chemical and molecular processes, regulating cellular homeostasis, humoral and cellular immune responses and being cofactors of many enzymes and antioxidant molecules. Moreover, metals have been exploited for industrial and economic purposes, increasing the risk of human exposure [1].

Several mechanisms, including metabolic and transport mechanisms and endogenous and exogenous antioxidant substances, are responsible for the protection of the body against the toxic effects of metals/metalloids. Therefore, the human organism has adapted significantly to live in contact with xenobiotics (Figure 1). However, some environmental agents, including metals and metalloids and the same essential oligoelements, can cause significant and serious acute and chronic toxic effects if consumed in certain doses [4]. Importantly, the toxicity threshold of essential trace elements derives from both their deficiency and their excess.

Several mechanisms are implicated in metal/metalloid toxicity, including the following: production of oxygen and nitrogen reactive species (ROS and RNS, respectively), interaction with thiol groups of proteins, incorrect protein folding and mimicry of the essential elements for intracellular transport and depletion of antioxidant enzymes, some of which require trace elements such as zinc, copper and manganese as cofactors. Proteins, membrane lipids and DNA are the molecular targets that are the most susceptible to the action of radical reactive species [4,5]. Under physiological conditions, radical species are readily neutralized by the organism’s antioxidant system, but when the radicals overcome this defense system, even by the action of some metals/metalloids, oxidative damage is induced.

The mechanism of protection against metal/metalloid toxicity can be lacking or insufficient in the case of genetic variants predisposed to autoimmune diseases, environmental and epigenetic factors. In susceptible individuals, with a genetic background predisposed to autoimmune disease, T cells falsely recognize the modified proteins as “non-self” and begin an autoimmune attack [6,7,8]. 

This review will give an overview of the literature relating to association and experimental studies exploring the possible influence of trace elements on autoimmune diseases, focusing its attention mainly on multiple sclerosis (MS) and type 1 diabetes (T1D). The increased incidence of these two diseases in the last 50 years cannot be explained only by genetic factors; environmental factors, such as trace elements and many others, might also affect immune regulation [9,10].

Since a great deal of evidence suggests that trace elements are important for autoimmune diseases and more in general for human health, and those covered below are mainly bio-accessible through food and water, this review also aims to point out the importance of homogenous legislative regulations about elements in drinking water. The achievement of this aim is very ambitious and can be realized only by studies conducted on large cohorts to define the reference ranges of trace elements in water in order to be applied worldwide.

## 2. Autoimmunity

The immune system consists of an innate part that is able to directly respond to foreign agents and an adaptive part that needs some time to develop an immune response. Moreover, mucosal surfaces play a key role in normal and dysfunctional immune responses [11]. All these elements may be affected by foreign agents, and the resulting interaction may lead to immunosuppression, immunostimulation and/or hypersensitivity and autoimmunity.

The failure to distinguish “self” from “non-self” is the basis of autoimmune diseases. Many autoimmune diseases arise when the tolerance towards self is lost due to a multifactorial interaction between genetic, epigenetic and environmental factors. Nearly 100 autoimmune diseases exist, some of which affect a single tissue (such as MS or T1D) or multiple organs (such as systemic lupus erythematosus, or SLE).

The natural selection of the body’s own molecules is carried out by the thymus during pregnancy and until puberty, when the senescence of the gland occurs. The thymus is an important primary lymphoid organ responsible for the maturation of T lymphocytes. Hematopoietic and lymphoid progenitors derived from bone marrow enter the thymus. Cells expressing the T cell receptor (TCR) are differentiated in CD4+ and CD8+ lymphocytes. In normal conditions, lymphocytes with potential reactivity against autologous antigens are sent to apoptosis (negative selection), while all other lymphocytes that respond tolerantly to autologous antigens are stimulated to mature (positive selection). After exiting the thymus, mature lymphocytes undergo a second selection process (peripheral tolerance), guaranteeing the elimination of the majority of self-reactive T cells [12]. Moreover, immature B cells expressing surface IgM, which recognize ubiquitous cell-surface antigens, are eliminated [12].

These selection processes are indispensable for the immune system to maintain the state of tolerance towards self. However, potentially self-reacting lymphocytes can escape these selection processes, entering circulation.

Genetic variants predisposed to the formation of less reactive T lymphocytes and therefore favoring their escape from the selection process and the generation of autoimmune responses have been discovered [13].

After the maturation process, CD8+ T cells circulate throughout the body, acquiring cytotoxic functions. They contribute to immunological homeostasis by killing cells that have been infected with viruses as well as cancer cells. On the other hand, CD4+ T cells are helper T cells with regulatory immunological functions and their role in autoimmunity is well known [14]. Helper T cells are divided into four main subpopulations, Th1, Th2, Th17 and regulatory T cells (Treg), whose imbalance effects an autoimmune condition [15].

Th1 cells differentiate under the influence of IL-12 and mainly produce interferon-γ (IFN-γ). IFN-γ activates macrophages, promoting the elimination of intracellular pathogens and thus supporting cellular immunity.

Th2 cells differentiate under the influence of IL-4. Th2 cells produce IL-4, IL-10 and IL-13, activating B cell proliferation and inducing antibody production, thus promoting humoral immunity.

Th17 cells are responsible for eradicating extracellular bacteria and fungi and activating neutrophils. Owing to their strong inflammatory properties, they are directly involved in autoimmunity and can also mediate chronic inflammation. It has been demonstrated that the pathogenetic mediators of many autoimmune diseases, such as MS, T1D, rheumatoid arthritis and psoriasis, are Th17 sub-populations beyond Th1 cells that are notably involved in autoimmunity [16,17].Th17 produce cytokines IL-17, IL-21, IL-22 and IL-23 [18]. Their induction and differentiation are complex, but, notably, it has been demonstrated that dietary factors such as salt (sodium chloride (NaCl)), both in vitro and in vivo, are strongly implicated in the initiation of exaggerated Th17 responses [19,20].

However, the role of Th17 has to be considered in association with Treg [18]. This specific cell population regulates immune and self-tolerance. In fact, T-regs are a highly specialized subset of T cells, responsible for optimizing the immune response; they are able to modulate the peripheral self-reactive T cell clones, including their activation and clonal expansion. T-reg cells participate in the induction and maintenance of immune tolerance through the recognition of autoantigens that are released from injured tissues, with subsequent modulation of the immune response, mediated by intimate cell–cell contact or via soluble cytokines [21].

In a number of autoimmune diseases, an altered Th17/Treg ratio has been shown [22], particularly a drastic drop in the T-reg population. In many cases, the successful treatment of a number of systemic autoimmune diseases by using different molecules and treatment approaches may also be linked to the restoration of the Th17/Treg imbalance and/or of cellular functional properties that had been shown for example in MS and SLE [23,24,25,26].

In some autoimmune diseases, such as MS, despite a normal frequency of Tregs being observed, it has been demonstrated that their suppressive function is substantially decreased in comparison to healthy subjects [27]. Interestingly, in murine models, increasing NaCl either in vitro or by diet markedly impairs Treg function [28].

Hence, there is evidence of profound interactions between simple elements such as NaCl and the more sophisticated immune mechanisms that, to our knowledge, have not been studied in detail or using the most innovative immunological techniques to investigate the role of trace elements.

Essential metals and trace elements can act as immunosuppressants or as immune adjuvants depending on the dose. Immunological effects of metals/metalloids include immunomodulation, autoimmunity and allergy. Immunomodulation consists of the ability of metals/metalloids to modify the production of cytokines in vitro and in vivo [29]. Adequate intake of micronutrients, including trace elements such as selenium, zinc, copper, and iron, supports an effective immune response by cytokines produced by Th1, avoiding a switch to a response mediated by Th2 cells. In fact, supplementation with these micronutrients can correct a Th2 cell-mediated immune response, inducing a response mediated by Th1 [10].

### 2.1. Multiple Sclerosis

Multiple sclerosis (MS) is an inflammatory, demyelinating and neurodegenerative disease of the central nervous system (CNS). The main sign of this disease is the formation of focal demyelinating lesions in the brain, optic nerve and spinal cord [30]. Lymphocytic infiltration causes inflammation and injury to the myelin sheaths of nervous fibers and consequently leads to the impaired ability of the nerves to conduct electrical impulses from and to the brain. Focal plaques can be observed around postcapillary venules and are characterized by the breakdown of the blood–brain barrier, leading to the increased trans-endothelial migration of activated leukocytes and eventually to oligodendrocyte loss and neuro-axonal degeneration [31].

The clinical manifestations and course of MS are heterogenous. However, the first symptoms are sensory and motor. Four clinical courses of MS have been defined [32]: relapsing–remitting MS (RRMS), which is the most common clinical form and is characterized by relapses occurring over time and alternating with neurological recovery episodes; primary progressing MS (PPMS), which affects 10–25% of patients and is characterized by a gradual disease progression and the absence of relapses; secondary progressing MS (SPMS), developed by most RRMS patients and characterized by a progressive and irreversible disability; progressive relapsing MS (PRMS), which is rare and characterized by acute episodes with or without recovery and a period of continuous disease progression between relapses.

MS predominantly affects young adults with an onset between 20 and 40 years of age. It has a higher female prevalence, with a sex ratio close to 3:1 (F:M) [33].

Recent data report 2.3 million cases of MS in the world. North America and Europe present the highest prevalence (140 and 108 per 100,000, respectively), while the lowest prevalence is registered in Sub-Saharan Africa and East Asia (2.1 and 2.2 per 100,000 respectively) [34]. Prevalence of MS increases according to latitude. An exception is represented by the Mediterranean island of Sardinia, with an overall age-adjusted MS prevalence of 330 per 100,000 [35].

MS risk factors include both genetic [36] and environmental factors such as vitamin D deficiency, Epstein–Barr virus (EBV) infection and alterations in gut microbiota [37,38,39]. Several studies have also shown a possible role of metals/metalloids in MS. Unbalanced serum levels of zinc, copper, manganese and iron have been associated with reduced anti-oxidative activity and MS [40,41]. In a murine model of experimental autoimmune encephalomyelitis (EAE), characterized by a dominant Th1/Th17 immunopathogenesis, zinc supplementation reduced EAE scores in C57BL/6 mice in vivo, reduced Th17 RORγT+ cells and significantly increased inducible iTreg cells [42]. Moreover, metals such as lead, copper and cadmium are also implicated in inducing the neurodegeneration and oxidative stress caused by an imbalance in their homeostasis and an imbalance between the formation of free radicals and their destruction by antioxidant molecules [43,44].

### 2.2. Type I Diabetes

Type I diabetes (T1D) is a chronic metabolic disorder caused by the selective loss of the pancreatic islet β-cells through an autoimmune mechanism. β-cell destruction leads to an insufficient production of insulin hormone and thus to increased blood glucose levels (hyperglycemia). Some evidence suggests a pathogenetic role of Th17 cells in the etiology of T1D due to an imbalance between Tregs and Th17, as for other autoimmune diseases [45,46,47].

T1D is associated with the development of autoantibodies against insulin, glutamate decarboxylase, insulinoma-associated protein 2 or zinc transporter 8 [48,49,50].

Insulin is the lifesaving permanent therapy. T1D patients must follow a structured self-management plan, including insulin treatment, monitoring of blood glucose levels, physical activity and a healthy diet [51].

The most common symptoms of T1D at onset are as follows: polyuria, polydipsia and polyphagia (which is often associated with weight loss, nausea, vomiting, muscle weakness, fatigue, visual disturbances and genital infections). Diabetic ketoacidosis is a serious life-threating complication, resulting from the mobilization of other metabolic sources of energy (ketones derived from fat) with the formation and accumulation of ketone bodies. Other chronic complications include retinopathy, neuropathy, nephropathy and cardiovascular diseases [52].

T1D is the most common form of diabetes in children, but it can develop at any age. In contrast to MS, TD1 predominantly affects males [53]. In 2019, 9.3% of adults aged between 20 and 79—amounting to 463 million people—have diabetes [54]. T1D accounts for about 10% of cases of diabetes worldwide, with 1.1 million children and adolescents under the age of 20 suffering from this disease.

The incidence of T1D is increasing worldwide. In Europe, T1D incidence shows a decreasing gradient from the Nordic countries to the Mediterranean countries. An exception is represented by Sardinia, whose population has the highest incidence in Europe, after Finland. Geographical variations in T1D incidence could be related to the peculiar genetic backgrounds of the most susceptible populations, such as Scandinavian and Sardinian populations. Genetic susceptibility is a clear risk factor for T1D. In particular, polymorphisms of class II HLA genes encoding DQ, DR and, to a lesser extent, DP represent strong genetic determinants of T1D [55,56,57,58,59]. Moreover, more than 50 non-HLA genetic factors that contribute to T1D risk have been identified in genome-wide association studies [60].

However, genetic risk factors are not sufficient for T1D onset but act in combination with environmental risk factors. Environmental and life-style changes have presumably contributed to the increased incidence observed in the last 30 years [61]. This hypothesis is supported by the fact that migrants acquire the same T1D risk as the native population in their new area of residence [62,63].

Environmental factors, such as dietary deficits, overweight, stress and infections affecting children in utero, at birth or during early childhood, play an important role in the development of T1D [61].

Several studies have also shown that anomalies in serum levels of metals are associated with T1D. Higher levels of copper have been detected in T1D patients compared to healthy controls [64]. Several studies have observed an association between chromium deficiency and diabetes [65,66]. Chromium deficiency has been associated with alterations in lipid, insulin and glucose metabolism [67]. Beneficiary effects of high levels of chromium supplement have been observed in diabetic patients [68]. Low concentrations of manganese impair insulin synthesis and secretion [69]. Furthermore, a correlation between the chemical elements and compounds present in soils and stream sediments in Europe and the incidence of complex autoimmune diseases, such as MS and T1D, has been suggested [70]. This study found significant positive correlations between barium and sodium oxide and the incidence of T1D and MS; meanwhile, negative correlations have been found for cobalt, chromium, copper, manganese and Zinc, elements typical of low-silicon lithologies, suggesting their potential protective effects against the onset of the two diseases [70,71]. These results are consistent with a Swedish study showing that a low zinc content in drinking water was associated with a higher risk of T1D in children [72].

## 3. Trace Elements

Trace elements are present in the human body in extremely small quantities of less than 0.01%. They are important for growth, development, maintenance and the recovery of health. They have various roles: some of them are essential components of enzymes, where they attract substrate molecules and facilitate their conversion into final products; others donate or accept electrons in the oxidation–reduction reactions necessary for the production and use of energy in the metabolism; others provide structural stability to some important biological molecules. Finally, some trace elements control important biological processes facilitating the binding of molecules to their receptors on the cell membrane, altering the structure or ionic nature of the membranes to regulate the access of certain molecules into the cell and inducing the expression of genes encoding for proteins involved in various vital processes.

Several studies report evidence of the possible involvement of trace elements in processes leading to an autoimmune response. For example, the causal relationship between mercury and immune diseases is well established and the mechanism has been hypothesized [7].

It has been discovered that mercury, nickel, cadmium, lead, aluminum and arsenic can exert immunotoxic effects through epigenetic mechanisms such as DNA methylation, post-translational modification of histones and miRNAs [73,74]. Furthermore, mercury, a possible risk factor for SLE, has been shown to induce the formation of anti-nuclear antibodies (ANA), hallmark of many autoimmune diseases, in mice [75].

The immunological effects of trace elements include immunomodulation, autoimmunity and allergy. These elements can act as immunosuppressants or as immune adjuvants. Their effects depend on the dose. In fact, their accumulation or deficiency can stimulate an alternative path that could induce the onset of disease. For example, low zinc levels and high levels of copper, manganese and iron participate in the activation of inflammatory responses and responses to oxidative stress induced by the ROS and RNS [40].

Interactions between different oligoelements may play an important role in metabolic disease onset. For example, copper deficiency anemia can develop in people who consume high doses of zinc over a long period of time [76]. Other interactions include the ability of selenium to reduce the toxicity of methylmercury, cadmium [77] and trivalent, pentavalent inorganic arsenic through the formation of a conjugated As-Se-glutathyl excreted with bile [78]. In vitro and in vivo studies have shown that small initial doses of cadmium are protective against subsequent high doses of cadmium [79,80]. In fact, cadmium induces the synthesis and storage of low molecular weight proteins (metallothioneins) in the liver and kidneys. Metallothioneins, which are rich in sulfur-containing amino acids, can bind to subsequent doses of mercury or cadmium, thus decreasing their toxicity [79]. High dietary intakes of calcium and magnesium can have a beneficial effect by reducing the gastrointestinal absorption of lead. However, calcium can also reduce the absorption of iron and zinc. Molybdenum can reduce copper retention [81].

The storage of trace elements in non-metabolically active sites or forms is another mechanism that allows the accumulation of elements at dangerous concentrations.

Furthermore, releasing trace elements from a storage site can play an important role in preventing deficiencies. In fact, current evidence suggests that the low affinity copper transporter (CTR2), located in the membranes of intracellular organelles, could serve to release copper from lysosomal deposits or from other vesicles towards intracellular spaces [82]. Similarly, zinc efflux transporters in the Golgi membranes and in the cell membrane play an important role in the homeostatic regulation of the element [82].

The World Health Organization (WHO) [64] classified trace elements into three groups based on their possible nutritional roles:

- potentially toxic elements, e.g., lead (Pb), cadmium (Cd), fluorine (F), mercury (Hg), arsenic (As), aluminum (Al), barium (Ba), lithium (Li), tin (Sn);
- elements of probable physiological importance, e.g., manganese (Mn), silicon (Si), nickel (Ni), boron (B), vanadium (V);
- essential elements, e.g., chromium (Cr), copper (Cu), zinc (Zn), selenium (Se), molybdenum (Mb), cobalt (Co), iodine (I).

The elements mentioned are introduced mainly with food.

A brief description of some elements, whose main sources are represented by food, water and diet, is reported below.

### 3.1. Potentially Toxic Elements

#### 3.1.1. Lead

Lead (Pb) is one of the most important toxic elements. It has been used for decades for many technological processes, including the production of paints, gasoline and aviation fuel. The previous use of carbonate and oxide lead in these products represents the main source of exposure to this metal that is not degradable and remains in the environment as dust, in the soil and paint in old houses.

Lead toxicity is based on molecular mimicry with cellular cations and the formation of ROS. It replaces zinc and calcium in proteins involved in different biological processes, consequently altering protein structure and function [83].

Lead passes through the placenta, determining an increase in the blood levels of the fetus nearly identical to that in the maternal blood [84]. Lead toxicity affects almost every organ in the body, but the central nervous system is particularly sensitive to lead effects in both children and adults. Learning deficits and behavioral problems are serious effects of lead poisoning in children, while in adults, neuropathies, chronic nephropathies, anemia, hypertension and toxicity related to the reproductive organs are of particular importance [85]. Another target of lead toxicity is the immune system. According to Fenga et al. (2017), lead enhances Th2 cell development, affecting Th1 cell proliferation and leading to high levels of IgE and inflammatory cytokines [86]. However, other authors have demonstrated that there are not changes in cytokine levels related to the Th1-, Th2- and Th17-mediated immune responses after short-term exposure to lead, in contrast to chronic exposure [87]. Therefore, lead alters Th cell functions, increasing the susceptibility to autoimmune diseases and hypersensitivity. For example, one study found a positive correlation between exposure to absorbable lead in soil and MS prevalence in Iran [43].

#### 3.1.2. Cadmium

Cadmium (Cd) is a toxic transition metal. Its industrial use was irrelevant up until 50 years ago. About 75% of the cadmium produced is used in batteries. It is also used as a pigment in paints and as a stabilizer in plastics. A potential source of cadmium is represented by the extraction of zinc and lead in mines, where lead is a secondary product. The natural presence of cadmium in zinc and lead deposits is well known.

The primary source of cadmium exposure is food, particularly cereals, leafy fruits and vegetables, crustaceans and liver and kidneys of animals, as well as contaminated drinks and cigarette smoke. Cadmium transplacental passage is not easy; however, since the absorption of micronutrients increases in pregnancy, cadmium absorption increases and accumulates at high concentrations. Cadmium exposure has been associated with nephrotoxicity, hepatotoxicity and effects on the immune system, bones and male reproductive physiology.

Several studies have shown that cadmium is an immunomodulator. It stimulates the production of Th2 cells. Stimulated macrophages and monocytes respond with the release of ROS and TNF α and production of the inducible nitric oxide (NO) enzyme synthase. Nitrogen oxides are known to regulate the proliferative response of lymphocytes [77]. Cadmium has been shown to inhibit glutathione reductase enzymes, implicated in the defense against free radicals, and the enzyme thioredoxin reductase, an oxidoreductase that reduces protein thiols and has an important role in regulating the redox state of cells [4]. Furthermore, an in vitro study has shown that low doses of cadmium stimulate the immune system, while higher concentrations inhibit immune responses [88]. This could be in agreement with the results of an Iranian study in which a high concentration of absorbable cadmium in the soil was associated with a lower prevalence of MS [43].

#### 3.1.3. Barium

Barium (Ba) constitutes about 0.05% of the Earth’s crust. Exposure to this element deserves particular attention because it is commonly found in surface waters and can be released into the environment by the natural breakdown of rocks and minerals or as polluting waste from industry and human activities.

The toxicity of barium-containing compounds depends on solubility, i.e., soluble salts are more toxic than insoluble salts because they can be absorbed through the skin or inhaled. A US study reported the presence of 10 ppm (parts per million) of barium in wheat and corn crops; milk, potatoes and flour were the main sources of barium in the American diet [89].

The mechanism of action of Ba involves blocking K⁺ efflux channels in the cell membrane, with a consequent increase in intracellular K+ levels and extracellular hypokalemia [90].

It can have an effect on skeletal muscles, smooth muscle and myocardial excitability and can lead to secondary respiratory paralysis and heart disease.

Although barium is found in low concentrations in the environment, the health consequences of chronic exposure have yet to be analyzed. A correlation study between geochemical data in Europe and the incidence of MS and T1D found a positive correlation between barium and sodium oxide present in soil and river sediments and these autoimmune diseases [70].

#### 3.1.4. Lithium

Lithium (Li) is a moderately abundant alkaline metal present in the Earth’s crust in an amount equal to 20 ppm. Lithium is easily absorbed by plants and its quantity varies widely, reaching 30 ppm in some cases. It is used for the production of alkaline batteries, soaps and large refrigeration and air conditioning systems. Lithium carbonate is used as an effective drug for the treatment of bipolar disorder.

Lithium can be absorbed by the inhalation of its aerosol and by ingestion. It was shown that treatment with lithium reduced MS symptoms in MS mouse models [91].

#### 3.1.5. Mercury

Mercury (Hg) is found in rocks in the Earth’s crust and occurs in coal and other fossil fuels. It is a common environmental pollutant. The most common sources of exposure are the consumption of fish and shellfish contaminated with methylmercury and the inhalation of mercury vapors in industrial environments—for example, during the preparation of dental amalgam [92].

The immunotoxic effects of mercury have been observed in humans and animal models. It has been shown that subtoxic doses of mercury may induce a systemic autoimmune syndrome in mouse models [93]. Low doses of mercury and methylmercury cause immunosuppression, reducing Th1 responses and increasing those of Th2 [94,95].

It has been suggested that mercury may have a role in accelerating or aggravating pre-existing systemic autoimmune conditions [96].

### 3.2. Likely Essential Elements

#### Silicon

Silicon (Si) is a very abundant element in the Earth’s crust, second only to oxygen, making up about 28% of the Earth’s weight. It is not found in nature in its free state but in the form of oxides and silicates. Small quantities (1–10 ppm) of silica and silicates dissolved or in colloidal suspensions are present in surface waters. It is possible to find silicon at different concentrations in various species of plants; for example, it is detectable at concentrations close to 1.2% in *Zea mays*.

In vivo studies have shown lipid peroxidation, oxidative stress, an increase in IgM, serum IgG and the presence of ANA in mice exposed to silica, supporting its role as an adjuvant of T lymphocytes and as a possible factor triggering autoimmune diseases such as SLE and glomerulonephritis [97].

An association between systemic immune diseases and occupational exposure to silica dust has been observed [98]. Silica-derived polymers such as silicone elastomer have been increasingly recognized as potential inducers of autoimmune and autoinflammatory (linked to innate immunity stimulation) syndromes [99,100].

### 3.3. Essential Elements

#### 3.3.1. Zinc

Zinc (Zn) is an essential trace element, ubiquitous in the environment and widely distributed in the body.

The dietary requirement for zinc is between 6.2 and 10.2 mg/day for women, between 7.5 and 12.7 mg/day for men and between 2.4 and 11.8 mg/day for children [101]. Rich sources of Zn in the diet are meat, milk, legumes, eggs, fish and cereals. Phytates (present in legumes, nuts and seeds), calcium and phosphates reduce zinc absorption. On the other hand, amino acids, picolinic acid and prostaglandin E2 can increase its absorption. Zinc needs increase up to two times during pregnancy and breastfeeding. In fact, zinc is lost in quantities of up to 2 mg per day until to 2 months after childbirth. Additionally, preterm infants require higher zinc levels due to inadequate deposits, reduced intestinal absorption and increased metabolic rate.

It has been observed that the fraction of zinc absorbed progressively decreases as zinc intake increases. This is due to the fact that Zn is an effective inducer of metallothionein synthesis, and when metallothionein in the intestinal cells is saturated, the absorption of Zn is reduced [101].

Zinc is predominantly found in muscles, bones, skin/hair, the liver and the pancreas. About 99% of zinc is intracellular and is distributed in the cytoplasm (50%), in the nucleus (30–40%) and in the cell membrane (10%), while the rest (about 1 mg/L) is bound to albumin in plasma (60–80%) [40].

Zinc is involved in a wide range of vital catalytic, structural and regulatory physiological processes and is required by more than 300 enzymes for their catalytic activation. It is involved in DNA, RNA and protein syntheses and is the cofactor of several transcription factors modulating the expression of zinc sensitive genes [102]. Furthermore, Zn binds to over 2500 proteins, maintaining their structural integrity and regulating their functions. It is one of the cofactors of the enzyme Cu/Zn superoxide dismutase (SOD), which plays a fundamental role in the removal of ROS and reduction of lipid peroxidation.

Zinc also plays an immune, anti-inflammatory and antioxidant role. It mediates innate immunity and influences acquired immunity by activating T lymphocytes and regulating the production of Th1 cytokines, B lymphocytes and antibodies. It is also used by macrophages for phagocytosis and cytokine production [102].

A recent meta-analysis has shown that low levels of zinchemia (in serum and plasma) are often observed in patients with autoimmune diseases [103]. Low zinc levels are known to contribute to immune defects associated with malnutrition [104]. In particular, a systemic zinc deficiency is associated with inflammation states, producing effects on the immune system. Interestingly, experiments conducted on cells, synoviocytes isolated from rheumatoid arthritis patients, have shown that the production of inflammation mediated cytokines IL-17/TNF in turn stimulates zinc uptake by the synoviocytes, thus increasing even more the inflammation, in a feedback loop between inflammation and zinc uptake [105].

As previously reported, Zn reduces the generation of ROS involved in the activation of NF-kB, a modulator of the immune response to infections whose dysfunction can cause autoimmune diseases and tumors. NF-kB inhibition results in the reduced generation of inflammatory cytokines and adhesion molecules [40].

Zinc deficiency induces thymic atrophy and reduces the activity of serum thymulin, a thymic zinc-dependent hormone necessary for the maturation and differentiation of T helper lymphocytes. The result is a decrease in Th1 cytokines, with a shift in activity towards Th2 lymphocytes and a reduction in the activity of natural-killer (NK) and cytotoxic T cells. Therefore, Zn deficiency induces an imbalance between Th1 and Th2 cell functions and between Treg lymphocytes and pro-inflammatory T cells, as well as the induction of Th17 lymphocyte activity, the main mechanisms that contribute to autoimmune disease pathogenesis. [40]. The proliferation of pre-activated human T cells and Th1/Th2/Th17 cytokine production may be suppressed by zinc aspartate [106].

A study has shown that the addition of Zn, in combination with probiotic complex and coenzyme Q1, to the diet of an animal model of arthritis suppressed the differentiation of Th17 lymphocytes [107]. Another study demonstrated that Zn suppressed Th17 development by inhibiting STAT3 activation in a mouse model of rheumatoid arthritis [108]. Zinc also induces a variety of other proinflammatory responses in T cells and B cells [109,110,111].

Studies in Sardinia and Sweden have shown that low concentrations of Zn in soils and drinking water were associated with a higher risk of T1D, suggesting that this metal has a protective role [71].

#### 3.3.2. Copper

Copper (Cu) is the third most abundant essential trace element in the human body, after iron and zinc, constituting 75–100 mg of the total quantity. The dietary requirements for copper are 1.6 mg/day for men, 1.3 mg/day for women and 1 mg/day for children [112].

Cu is widely distributed in nature and is found at a concentration of about 55 ppm in the Earth’s crust (77). It is almost always found in the form of minerals, such as sulphides, oxides, carbonates, silicates or, more rarely, in their native states. The most abundant minerals are copper and iron sulphides (chalcopyrite).

Food, drinks and water are the main source of exposure of the population to copper sulphate. Copper is contained in greater quantities in meat, liver and kidneys, in cereals, mollusks and in some fruits (avocado, walnuts, hazelnuts and dried grapes).

Copper exists in two oxidation states, as Cu (I) or Cu (II), and this ability to gain or lose an electron is at the basis of its role in the energy transfer processes in biological systems and in cellular respiration.

Many enzymes require copper as a cofactor, particularly those involved in iron metabolism, in the synthesis of neurotransmitters, in energy metabolism and in the cross-linking of collagen and elastin.

Copper deficiency symptoms have been observed in a X-linked recessive disease due to mutations of the copper transporter ATP7A. They include anemia, hypercholesterolaemia, metabolic syndrome, reduced glucose tolerance, hypopigmentation of the skin and hair, leukopenia, neutropenia, myelodysplasia and, in most patients, neurological effects, most commonly due to neuromyelopathy [113,114]. It has been hypothesized that anemia associated with copper deficiency is due to defective iron mobilization, resulting from reduced ceruloplasmin activity [114]. This enzyme, with its ferroxidase action, is fundamental for the transformation of Fe^2^⁺ to Fe^3^⁺, an indispensable step for the incorporation of iron into the circulating transferrin to avoid the toxicity of the free metal involved in the production of free radicals. Furthermore, copper deficiency has been associated with changes in immune and bone function. In particular, a reduced number of leukocytes and neutrophils and reduced antioxidant activity of Cu/Zn SOD, as well as the presence of lipid peroxidation, have been detected [113].

Since copper is involved in the synthesis of the myelin sheath, its deficiency can potentially cause myelopathy [115]. Moreover, copper has a regulatory role in cell growth and in maintaining homeostasis in the immune system. In particular copper sulphate seems to exert beneficial effects in T1D mouse models, both by directly reducing the amount of free radicals and by lowering blood glucose levels [116].

#### 3.3.3. Chromium

Chromium (Cr) is an essential nutrient involved in protein, lipid and carbohydrate metabolism. The dietary requirement for chromium is 20–35 μg/day [117]. Chromium is naturally present in trivalent, Cr (III), and hexavalent, Cr (VI), forms. Cr (VI) has been extensively used in the paint, steel manufacturing and leather industries. The association between Cr (VI) toxicity and lung cancer in stainless workers is well established [118]. Moreover, it has been demonstrated that Cr (VI) induces oxidative stress by increasing the production of ROS [119].

On the other hand, Cr (III) salts have been shown to possess beneficial effects as nutritional supplements in animals and humans [120].

Chromium deficiency has been observed in diabetic patients receiving chronic total parenteral nutrition. Chromium supplementation resulted in improved glucose tolerance [121].

## 4. Legislation of the Elements in Water and Food

The legislation of the European Union and of the Member States aims to protect human and animal health and to restrict environmental pollution.

Metals and metalloids are regulated for many media, and some of them are directly in contact with humans: food, drinking water, surface water and swimming pool water and groundwaters. The regulations also set limits for soils and terrains, feed and waste.

The correct disposal of municipal and industrial waste is of particular importance, and the Sixth Ministerial Conference on Environment and Health of 53 countries in the European area of the World Health Organization (WHO), held in Ostrava, Czech Republic (13–15 June 2017), included the issue of contaminated sites among the public health priorities for the first time.

In particular, in the Ostrava Declaration, the States of the European Union are urged to adopt programs and actions to prevent and eliminate adverse environmental and health effects, costs and inequalities relating to the management of waste and contaminated sites.

In Europe, the presence of around 342,000 contaminated sites has been estimated, of which only 15% have undergone environmental remediation.

The contamination of these areas refers in particular to both soil and water, for which industrial activities and the management and treatment of industrial waste represent the main sources of pollution.

Although the containment of industrial emissions has improved in recent decades, the industrial sector is still responsible for the presence of significant quantities of pollutants in water, air and soil, as well as for the production of waste.

To limit risks to humans and the environment, the European Union defines specific legislative limits for each metal/metalloid.

- **Directive 98/83/CE**, relating to the quality of water intended for human consumption [122,123,124], was transposed in Italy, with the emanation of Legislative Decree No. 31/2001, which defines “water devoted to human consumption” as
  - all treated or untreated waters, intended for drinking, culinary or food preparation or for other domestic uses, regardless of their origin, whether they are supplied through a distribution network, by means of tanks, in bottles or containers;
  - all waters used in a food business for the manufacture, treatment, conservation or placing on the market of products or substances intended for human consumption.

With the Italian Legislative Decree no. 31/2001, it is established that waters intended for human consumption “must not contain microorganisms and parasites, nor other substances, in quantities or concentrations that represent a potential danger to human health”.

- **Directive 2003/40/CE** determines the list, the concentration limits and the labeling indications for the elements and compounds of natural mineral waters, as well as the conditions of use of ozone-enriched air for the treatment of natural mineral waters and spring waters. This Directive has been transposed in Italy with the Ministerial Decree of 29 December 2003. It imposes less restrictive limits on some elements and compounds of mineral water, especially with regard to those substances that can be dangerous to health.

It includes the following mandatory mentions on the label and in clearly visible characters (but this regulation is often disregarded):

○ “water subjected to an ozone-enriched air oxidation technique”, if this technique is used to remove iron, manganese, sulfur and arsenic residues;
○ “it contains more than 1.5 mg/L of fluorine: regular consumption by infants and children under the age of 7 is not appropriate”.

It should be pointed out that there are some discrepancies between the two regulations; in fact, some concentration values in water intended for human consumption deviate significantly from the values indicated for natural mineral waters. For example, for cadmium, the concentration in water devoted to human consumption must not exceed 5.0 μg/L, while in natural mineral waters, a concentration of 3.0 μg/L is allowed for the same element. Different values are also observed for boron, copper, manganese and fluorides.

The high concentration of a certain element in waters devoted to human consumption cannot be considered, at the same time, dangerous for human health if those waters derive from an aqueduct and safe if those waters came from a mineral source: if a certain dose of an element is harmful to health, it will always be harmful, regardless of the “product category” of the water in which it is found. Therefore, the lawmaker needs to standardize data for water devoted to consumption, including groundwater, which is subject to a third legislation (D.Lgs. 152/06). Furthermore, the national regulations mentioned above do not take into consideration other elements, well known for their toxicity. For these elements (including thorium and uranium), although their effect on health through acute and/or chronic exposure is known, WHO and national directives do not provide a threshold value due to their generally low concentration in natural waters, not requiring verification of any standard [124].

Italy, thanks to its geology and morphology, is among the richest countries in terms of sources of natural mineral waters in the world and, according to a 2018 Censis survey, it is one of the major producers and consumers of mineral water, with 350 registered sources and an average consumption rate of 206 L per capita per year. Therefore, careful control of its chemical-physical parameters is very important.

Various elaborations were carried out to compare the concentration analytical data of different elements and compounds, with those relating to the lithology, ore deposits and the anthropization degree (e.g., the presence of industries, urban agglomerations etc.) of the reference areas of the various sources.

In some cases, certain elements have been identified that, due to their concentrations, could have negative effects on human health despite having a purely natural origin, due to the geological characteristics of the environments in which the waters are accumulated or in which the waters transit, before flowing on the surface and/or being collected. However, the high values of these toxic elements, although not caused by anthropogenic activities, should in any case be reduced during production, if distributed through bottling as mineral waters or through tap. This is also the case for uranium, found in tap water in the autonomous province of Bolzano, where the natural presence of this element in the environment and water was detected, recording concentrations far higher than the threshold indicated by the Environmental Protection Agency (EPA).

- **Regulation n. 1881/2006 CE** defines the maximum values of some contaminants in food.
- **Regulation CE 333/2007** deals with the methods of sampling and analysis for the official control of the maximum values of metals/metalloids;
- **Council Directive 96/23/CE** covers the surveillance of residues of chemical elements in food of animal origin;
- **Regulation (UE) n. 1169/2011** details the provision of food information to consumers: mandatory information on the label;
- **Regulation (UE) N. 432/2012** relates to the compilation of a list of permitted health claims on food products, for vitamins, minerals and other substances, e.g., “Zinc contributes to the normal function of the immune system”;
- **Directive CE 2009/54** details the use and marketing of natural mineral waters.
  This directive does not apply
  (a) to waters which are medicinal products within the meaning of Directive 2001/83/EC of the European Parliament and of the Council, of 6 November 2001, on the Community code relating to medicinal products for human use;
  (b) to natural mineral waters used for healing purposes at the source in thermal or hydrothermal establishments.

“All indications that attribute, to a natural mineral water, properties for the prevention, treatment or healing of a human disease are prohibited. Member States may authorize the terms “stimulates digestion”, “may promote hepatobiliary functions” or similar terms””.

- **D. Lgs. N. 28, 15/02/2016**: Implementation of Council Directive 2013/51/EURATOM, of 22 October 2013, which establishes requirements for the protection of the population’s health with regard to radioactive substances present in water devoted to human consumption.

Inside, the legislation defines as a radioactive substance “any substance containing one or more radionuclides whose activity or concentration cannot be neglected for radiation protection purposes”. In this decree, most radionuclide compounds are considered under a single heading and a reference threshold value equal to the overall exposure called the “indicative dose” (the effective dose committed for one year of ingestion resulting from all the radionuclides, of natural and artificial origin, present in water intended for human consumption, with the exception of tritium, potassium-40, radon and short-lived radon decay products).

This decree is not applied to mineral waters or medicinal waters, confirming again the strong differences in the regulation of these products compared to drinking water.

A clarification is necessary: although the danger from radioactivity exposure deriving from uranium is included in this decree, at present there is no regulation in Italy that regulates the reference concentration value for its toxicity (as a chemical element) if taken through water, even though there are well-known cases of its presence in mineral waters from Italian springs (mainly from granite rock springs).

Although its long-term effects are not currently known, excessive uranium intake is linked to the onset of kidney disease. Its effect on fertility and its role as an endocrine disruptor at concentrations lower than those suggested by the EPA are currently being investigated.

- **Decree 10/02/2015**: Assessment criteria of the characteristics of natural mineral waters.

The analyses for the quality assessment of mineral waters require only the analysis of the organoleptic properties and a restricted set of parameters (together with the non-exceeding of the values already provided by the DM 29/12/2003 indicated in Table 1).

Among these, the following are included: silicon, bicarbonate, chlorides, sulfates, sodium, potassium, calcium, magnesium, iron, phosphorus, strontium, lithium, aluminum, bromides and iodides.

Although verification of these parameters is requested, their publication on the bottle label is not mandatory and neither is the analysis and verification of those potentially toxic and naturally present elements in the environment.

At the conclusion and integration of what has been reported so far, it is necessary to emphasize that the water is not the same everywhere [125,126,127,128], but it is inextricably linked to the local geological conditions, and, on this basis, the regulations should be calibrated. In fact, it is often difficult to be able to intervene in the sanitization of water without having the origin data of the elements present in it, as each element is characterized by its own behavior and bio-availability, dependent on a series of very complex variables to be evaluated. It should be noted that the technical solutions currently available for sanitizing water are generally of little effectiveness, at least on a domestic scale, since they involve the presence of devices that require special management and particular maintenance and, in any case, these devices are only available to a restricted part of world population. In short, in order to be used in a widespread and effective way, these treatments should not require technologies which are too sophisticated, fragile and/or expensive. The ideal outcome would be to have a technology that allows the use of the resource, of any origin, ensuring the correct balance of the substances contained through the self-regulation of the device. This technology is under study [129], although still in the preliminary phase, but this allows us to be moderately optimistic about a solution in the near future.

## 5. Conclusions

The life of organisms is strongly influenced by trace elements, by their bioavailability in the environment and by their homeostasis, which must be maintained in the organism itself. Some elements can be toxic even in very small traces, while others are essential for cellular functionality. In addition to environmental factors such as diet, drugs and infections, even trace elements like those covered in this review, as well as others, can be decisive in maintaining or breaking immunological tolerance or in silencing or amplifying autoimmunity in genetically predisposed subjects. It is therefore desirable that greater attention is paid by the legislative bodies to this matter, and correct knowledge of health workers regarding both the potential toxic and beneficial effects of these elements in human health is essential.

As aforementioned, there is no doubt that water often presents critical issues that not even the most recent regulations consider. In fact, they report different thresholds for the same element based on inadequate assumptions. The careful protection of and safe access to healthy water could have strong economic implications, which are difficult to evaluate, deriving from the vital importance of water for health. In particular, autoimmune diseases have a huge impact on the world economy since they are chronic diseases requiring treatment throughout life. The stochastic effects—for example, deriving from the excess or deficiency of certain elements—represent a highly underestimated criticality which could significantly affect the general costs of public health. It is therefore imperative that the legislation fills this legislative gap as soon as possible.

## Figures and Tables

**Table 1:** Threshold concentration values for mineral water and water devoted to human consumption.

Threshold Concentration Values for Mineral Water and Water Devoted to Human Consumption							
	DM 29/12/2003 (Italy) Mineral Water	D.L. 31/2001 (Italy) Water Devoted to Human Consumption	Directive EU 2003/40/CE Mineral Water	Directive EU 1998/83/CE Water Devoted to Human Consumption	EPA (USA) Guidelines	WHO Guidelines	D. LGS. 152/06 (Italy) Groundwater
Ec (μS/cm)	-	2500 (g.l.)	-	2500 (g.l.)	-	-	-
pH	-	≥6.5–9.5 (g.l.)	-	≥6.5–9.5 (g.l.)	≥6.5–8.5	-	-
Aluminum (μg/L)	-	200 (g.l.)	-	200 (g.l.)	-	200	200
Ammonium (mg/L)	-	0.5 (g.l.)	-	0.5 (g.l.)	-	-	<0.05
Antimony (μg/L)	5	5	5	5	6	20	5
Arsenic (μg/L)	10	10	10	10	10	10	10
Barium (μg/L)	1000	-	1000	-	2000	1300	-
Beryllium (μg/L)	-	-	-	-	4	-	4
Boron (μg/L)	5000	1000	-	1000	-	2400	1000
Cadmium (μg/L)	3	5	3	5	5	3	5
Chlorides (mg/L)	-	250 (g.l.)	-	250 (g.l.)	-	250	-
Chromium (μg/L)	50	50	50	50	100	50	50
Iron (μg/L)	-	200 (g.l.)	-	200 (g.l.)	200	-	200
Fluorides (mg/L)	5 (1.5 *)	1.5	5	1.5	4	1.5	1.5
Phosphorus (mg/L)	-	-	-	5	-	-	-
Lead (μg/L)	10	10	10	10	15	10	10
Manganese (μg/L)	500	50 (g.l.)	500	50 (g.l.)	-	400	50
Mercury (μg/L)	1	1	1	1	2	6	1
Molybdenum (μg/L)	-	-	-	-	-	70	-
Nickel (μg/L)	20	-	20	20	-	70	20
Nitrates (mg/L)	45 (10 *)	50	50	50	10	50	-
Nitrites (mg/L)	0.02	0.5	0.1	0.5	1	3	0.5
Copper (μg/L)	1000	1000	1000	2000	1300	2000	1000
Selenium (μg/L)	10	10	10	10	50	40	10
Sodium (mg/L)	-	200 (g.l.)	-	200 (g.l.)	-	200	-
Sulphates (mg/L)	-	250 (g.l.)	-	250 (g.l.)	-	500	250
Thallium (μg/L)	-	-	-	-	0.5/2	-	2
Uranium (μg/L)	-	-	-	-	30	30	-
Vanadium (μg/L)	-	140	-	-	-	-	-
Zinc (μg/L)	-	-	-	-	-	3000	3000

(*) Legal threshold concentration value for water devoted to consumption by infants; (g.l.) guidelines.
